# Advances in anti-BRAF therapies for lung cancer

**DOI:** 10.1007/s10637-021-01068-8

**Published:** 2021-01-21

**Authors:** Giandomenico Roviello, Alberto D’Angelo, Marianna Sirico, Matteo Pittacolo, Felipe Umpierre Conter, Navid Sobhani

**Affiliations:** 1 Department of Health Sciences, Section of Clinical Pharmacology and Oncology, University of Florence, viale Pieraccini, 6, 50139 Florence, Italy; 2 Department of Biology & Biochemistry, University of Bath, Bath, BA2-7AX UK; 3 Multidisciplinary Operative Unit of Mammary Pathology and Translational Research, ASST of Cremona, 26100 Cremona, Italy; 4 Department of Surgery and Cancer, Imperial College London, London, UK; 5 Department of Medicine, Section of Epidemiology and Population Sciences, Baylor College of Medicine, Houston, TX 77030 USA

**Keywords:** BRAF, Lung cancer, V600

## Abstract

Non-small cell lung cancer (NSCLC) is one of the most frequent causes of mortality in the western world. v-raf murine sarcoma viral oncogene homolog B (BRAF) is a member of the Raf kinase family and plays a critical role in cellular growth, proliferation, and differentiation through the mitogen-activated protein kinase pathway. The incidence of BRAF mutations in NSCLC is low, accounting for 0–3% of all cases of lung cancer. Given the results obtained in metastatic melanoma, several studies have reported the efficacy of anti-BRAF therapies in NSCLC treatment. In this review, we describe changes in the landscape of BRAF-mutated lung cancer treatment and analyze insights from major clinical trials in the context of future therapeutic prospects.

## Introduction

Lung cancer is the most frequent cause of mortality worldwide, with an estimated 40% of cancer-related deaths [1]. Non-small lung cancer (NSCLC) accounts for approximately 85% of lung cancer cases and is divided into nonsquamous (70%), squamous (25%), and unspecified (5%) histological characteristics [2]. Between 2008 and 2014, the median overall survival (OS) of patients with NSCLC has been historically poor, with a 5-year survival of 24% for all patients and 5.5% for those with distant metastases [3]. Lung cancer is a heterogeneous disease composed of different clonal sub-populations harboring different molecular characteristics [4]. Over the past two decades, important advances in the treatment of NSCLC have been achieved, thereby increasing our understanding of the disease biology and tumor progression mechanisms. A better understanding of the biology of lung cancer has led to the development of novel biomarker-targeted therapies and heralded the era of precision medicine.

The most common genetic alteration in NSCLCs is associated with the epidermal growth factor receptor (*EGFR*) genes. This mutation, present in approximately 10–13% of Caucasian patients with lung cancer, has a role in the early phase of tumor initiation and represents a potential target for targeted therapy [5]. Other important mutations are related to anaplastic lymphoma kinase gene (*ALK*) rearrangements and the c-ros oncogene 1 (ROS1). Recently, additional molecular alterations have been identified, including amplification and mutations in the hepatocyte growth factor receptor (*MET*) and human epidermal growth factor receptor 2 (*HER2*) genes, rearrangements in the rearranged during transfection gene (*RET*), mutations in v-raf murine sarcoma viral oncogene homolog B (BRAF), and, according to the latest reports, alterations in the neurotrophic tropomyosin receptor kinase gene (*NTRK*). All these mutations are potentially druggable targets [6]. BRAF is a member of the Raf kinase family and plays a critical role in cellular growth, proliferation, and differentiation through the mitogen-activated protein kinase (MAPK) pathway [7]. This gene is altered with high mutational rates in various types of cancer, such as hairy cell leukemia (100%), melanoma (>60%), and papillary thyroid cancer (>50%) [8]. The most common BRAF mutation occurs at the level of T1799 transversion in exon 15, leading to the substitution of valine for glutamic acid (V600E) [9]. This alteration leads to constitutive activation of B-Raf kinase and the subsequent signal transduction to the MAPK/extracellular signal-regulated kinase (ERK) cascade, resulting in a 10-fold increase in BRAF activity compared to that in the wild-type (WT) protein [10]. Mutant BRAF is a prototype of the driver oncogene; its inactivation leads to cancer cell apoptosis, thereby indicating the existence of an acquired dependency of tumor cells on this mutant form of BRAF [11]. Given the results obtained in metastatic melanoma, Planchard et al. assessed the antitumor activity of dabrafenib monotherapy in BRAF (V600E)-mutant NSCLC in a study published in 2016. In this phase II open-label study, 84 patients were enrolled, of which 78 were treated and 6 were untreated. With a median follow-up of 10.7 months, an overall response rate (ORR) of 33% with a disease control rate of 58% was reported for dabrafenib monotherapy in the 78 previously treated patients with metastatic BRAF^V600E^-mutated NSCLC. In patients treated with one to three previous lines of therapy, median progression-free survival (PFS) and duration of response were 5.5 and 9.6 months, respectively, and median the preliminary median OS was 12.7 months [12]. This was the first prospective trial of BRAF inhibition to focus on BRAF^V600E^-mutated NSCLC. Primarily, antitumor activity of BRAF inhibitors (BRAFi) was shown only in some isolated case reports [13]. Human et al. studied the activity of vemurafenib in patients with BRAF^V600E^ mutation in a phase II basket trial in 2015, and reported a ORR response rate of 42% and a median PFS of 7.3 months in the cohort with NSCLC [14]. Given these results, in 2016, the Food and Drug Administration (FDA) approved this drug for treatment of advanced NSCLC in patients harboring BRAF^V600E^ mutation [15], and a molecular-level targeted approach was adopted for BRAF-mutant NSCLCs. In this review, we describe the changes in the landscape of BRAF-mutated lung cancer treatment and provide insights and future perspectives by analyzing major clinical trials.

### Clinical characteristics of BRAF-mutated NSCLC

In NSCLC, the incidence of BRAF mutations is low, accounting for 0–3% of all cases of lung cancer. Since BRAF acts as an oncogene in NSCLC, driver mutations in *BRAF* are mutually exclusive from *EGFR* mutations or *ALK* rearrangements [16]. Even if The Cancer Genome Atlas reported a 3% mutation rate in squamous cell lung cancer, this alteration is almost completely confined to the adenocarcinoma subtype [17]. BRAF^V600E^ mutation represents the most frequent BRAF mutation in lung cancer, accounting for approximately 50% of BRAF-mutant NSCLC, whereas fewer mutations have been identified at the G469A and G594G sites [18]. The occurrence of BRAF mutations has been reported to be lower in Asian (1.3%) populations than in Caucasian ones (3%), probably because of ethnic differences and the high frequency of EGFR mutations in Asian women with lung adenocarcinoma [19]. This mutation does not result in specific differentiation with respect to clinical features. However, some differences do exist between V600E and non-V600E mutants. BRAF^V600E^ mutations have been reported to be more frequent in female patients and could not be correlated to smoking history, whereas non-V600E mutations were more likely to arise in males with a history of smoking [20]. Concerning the prognostic significance of BRAF mutations in NSCLC, as in colorectal [21] and papillary thyroid cancer [22], BRAF^V600E^-mutant tumors are associated with poor prognosis compared to the non-V600E ones [23]. Specifically, Marchetti et al. in his retrospective study of 1046 NSCLCs harboring BRAF mutations indicated an association between V600E mutations and T status, N status, and pathological state, and a significantly shorter patient DFS and OS than those without these mutations [24]. This prognostic impact could be attributed to an aggressive histologic architecture such as non-mucinous adenocarcinoma, showing micropapillary features, acinar growth, and solid growth [25]. In contrast, non-V600E mutations in BRAF were not related to any specific histological features or prognosis [26].

### Molecular pathway of BRAF

**Fig. 1:**
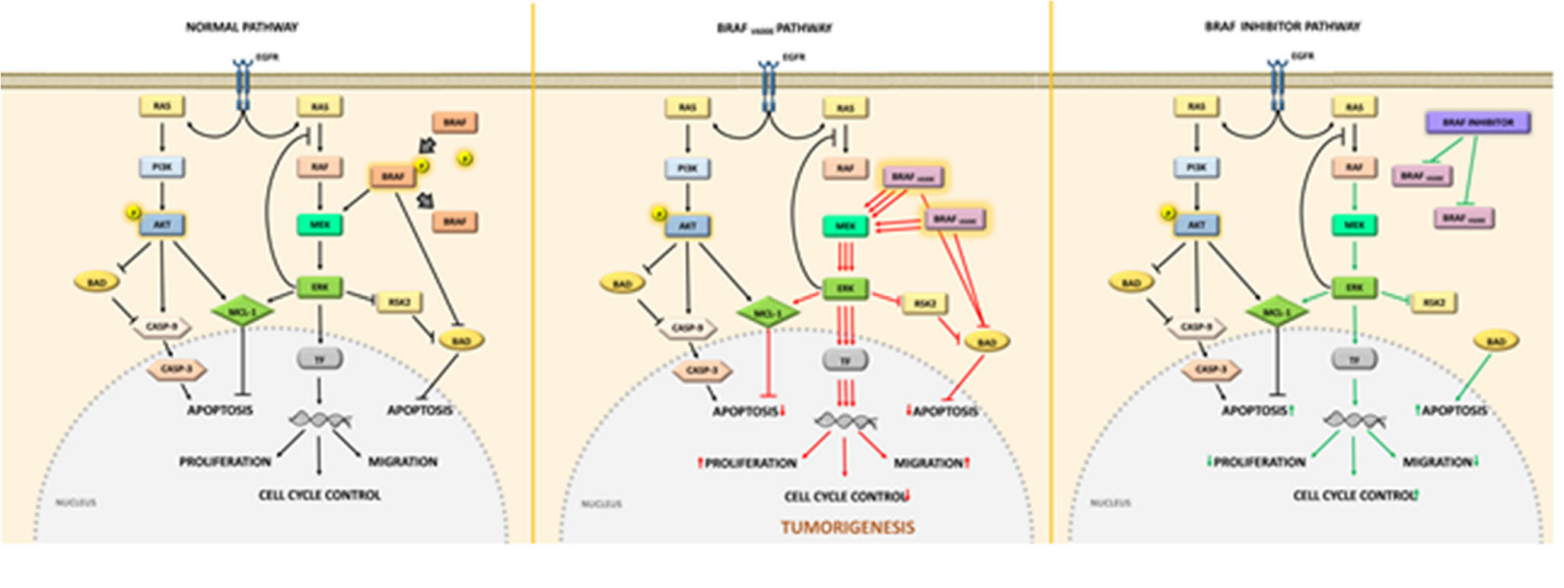
Normal functional MAPK/ERK pathway in wild-type (WT) BRAF and its alteration in the presence of BRAF^V600E^ mutation and the mechanism of action of drugs targeting the mutation. Normal functional MAPK/ERK pathway is activated after extracellular signaling, leading to a response in cell cycle control, proliferation, and cellular migration. The BRAF^V600E^ mutation induces a self-sustained constant activation of the MAPK/ERK pathway, thereby inhibiting controlled cellular death by apoptosis via indirect regulation of BAD. The BRAF^V600E^ pathway drastically increases the basal levels of proliferation, consistent with oncogenic development. Therefore, the BRAF^V600E^ mutation could be targeted for treatment with specific inhibitors, which results in an increase in apoptotic activity

BRAF belongs to the family of serine-threonine protein kinases, and is an important effector molecule for the MAPK/ERK signaling pathway (Fig. 1). Somatic mutations in BRAF, leading to the V600E variant, alter two main regions of the peptide, by disrupting the glycine-rich P loop and its variant domain of the kinase segment. The conversion between active and inactive states in WT BRAF is carried out through activation of the inhibitory effect triggered by the glycine-rich P loop, which is extremely important for the incorporation of signal transduction provided by RAS [27, 28]. Because of the phosphomimetic characteristics of the V600E mutation of BRAF, the glutamate residue interacts directly with the glycine-rich P loop by blocking its function as an inhibitory regulator, thereby leading to a constant state of spontaneous activation of BRAF and initiating a cascade of events that requires no signalization from external stimuli on EGFR (Fig. 1). The resulting over-stimulated proliferation of the cell renders it independent of the influence of external growth factors [29, 30]. The inactivation of the control mechanism significantly increases the basal activity of the cell (up to 10 fold) and is carried over to the next cell division cycles, leading to a strong oncogenic pattern [8, 31]. Figure 1 demonstrates the mechanism by which V600E mutation overloads the MAPK pathway, decreasing the activation of apoptotic mechanism regulated by BAD and through caspase cascade events. High activity of ERK leads to an increase in the proliferation process and an increase in cellular migration, without EGFR-mediated initiation triggered by the presence of external stimuli [32]. This robust pathway also implies that selective inhibition of BRAF^V600E^ disrupts the over-stimulated pathway, allowing the repair mechanisms associated with damaged cyclins to trigger apoptosis [33].

### BRAF mutations

Approximately 200 BRAF-mutant alleles have been identified in human tumors, which could be further classified into three classes based on kinase activity, RAS-dependence, and dimerization status. Class 1 BRAF mutations (V600E/D/K/R) are the most frequently identified in solid tumors; these mutations lead to a strong activation of BRAF kinase activity and a constitutive activation of the MAPK pathway [34]. Class 2 BRAF mutations are divided into high or intermediate kinase activity based on MAPK pathway activation, and these mutants signal as constitutively active dimers. Both class 1 and class 2 BRAF mutations are RAS-independent and, therefore, resistant to RAF inhibitors [35]. Class 3 BRAF mutations have low or absent kinase activity; they are related to ERK-mediated feedback, and their activation is RAS-dependent. These mutants are not independent drivers and require upstream activation of the MAPK pathway; therefore, this class of mutations frequently coexist with RAS mutations or NF1 loss. Hence, a potential therapeutic strategy is the blocking of upstream RAS signaling [36].

A recent publication showed that, in addition to these three class of mutations, missense mutations, deletions, and a number of BRAF fusions called “mutations of unknown functions” have been identified [37]. For example, the BRAF fusion gene has been found in melanomas, prostate and gastric cancer, and in 85% of astrocytic pilomcytomas [38]. These mutations are single events, not evidenced in the largest database of solid tumors, and frequently coexist with other driver mutations [39]. Due to their low frequency of occurrence, these could be passenger mutations, thereby accounting for their lack of response to MAPK therapies. Therefore, further investigation of these rare BRAF mutants is warranted to distinguish BRAF drivers from passenger mutations.

### FDA-approved anti-BRAF inhibitors

PLX4032, also known as vemurafenib, is a potent inhibitor of the BRAF mutant family. The name “vemurafenib” is derived from its ability to inhibit V600E-mutated BRAF [40]. It was approved by the FDA in 2011 after results from a phase III trial (BRIM-3), which showed improved OS and PFS rates in patients with BRAF^V600E^-mutated melanoma [41]. In addition, vemurafenib has been approved by the FDA for the treatment of unresectable advanced melanoma [42]. Since 2017, it has also been approved for the Erdheim-Chester disease containing the BRAF^V600E^ mutation [43]. PLX4032 is known to promote the apoptosis of mutated cells in a dose-dependent manner. Specifically, it interrupts the BRAF/MEK step in the BRAF/MEK/ERK pathway. PLX4032 is specifically active in BRAF-mutated cell lines [44]. The most common side effects in patients receiving vemurafenib are arthralgia, skin rash, nausea, photosensitivity, fatigue, pruritus, palmar-plantar dysesthesia, and cutaneous squamous cell carcinoma. As described by Flaherty et al., most PLX4032-related side effects appear to be proportional to the dosage used and the time of exposure [45].

Dabrafenib (GSK21188436) is a small molecule inhibitor of the BRAF-mutant kinase family. It is used in monotherapy or in combination with trametinib for the treatment of unresectable or metastatic BRAF^V600E^-mutated melanoma, advanced BRAF^V600E^-mutated NSCLC, and BRAF^V600E^-mutated locally advanced or metastatic ATC [46]. Dabrafenib is a potent ATP-competitive inhibitor of BRAF kinase and is highly selective for mutant BRAF in kinase panel screening, cell lines, and xenografts [47]. Since 2013, it has been approved by the FDA for the treatment of advanced melanoma, on the basis of the results of a phase III trial (NCT01227889), in which an improved PFS compared to that with dacarbazine was reported [48]. The most common side effects seen with dabrafenib monotherapy are hyperkeratosis, headache, pyrexia, and arthralgia [49].

Trametinib (GSK1120212) is a type III, reversible allosteric, non-ATP competitive inhibitor of both MEK1 and MEK2. It binds within the cleft between the small and large lobes of the kinase, adjacent to the ATP binding pocket, so that both the ATP and the allosteric inhibitor can bind simultaneously to the kinase [50]. Trametinib can be used for monotherapy; however, in most cases, it is used in combination with dabrafenib for the treatment of adults with unresectable or metastatic melanoma harboring BRAF^V600E^ mutation [51]. Trametinib is well tolerated, and the spectrum of side effects is consistent with that of MEK inhibitors. Skin-related toxicity, diarrhea, and most common side effects such as arthralgia, rash, headache, and fatigue are adequately managed with supportive care alone. Cardiac, ophthalmologic, and hepatic events are uncommon, and these have been reported to be reversible on interruption of trametinib treatment [52].

Encorafenib (LGX818), a new generation BRAFi, targets key enzymes in the MAPK signaling pathway [53]. It acts as an ATP-competitive RAF kinase inhibitor, decreasing ERK phosphorylation and downregulating cyclin D1 [54]. Encorafenib exhibits a more prolonged pharmacodynamic activity than other approved BRAFi molecules [55]. On June 27, 2018, FDA approved the combination of encorafenib and binimetinib (an anti-MEK1/2 protein kinase inhibitor) for treatment of patients with unresectable or metastatic melanoma with a BRAF^V600E^ or BRAF^V600K^ mutation, based on insights from an FDA-approved test. The approval was based on a phase III randomized, active-controlled, open-label, multicenter trial (COLUMBUS), which enrolled 577 BRAF^V600E^ or BRAF^V600K^ mutation-positive patients with unresectable or metastatic melanoma [56]. In this trial, the combination of encorafenib and binimetinib resulted in a longer PFS than vemurafenib. The most common (≥25%) adverse reactions in patients receiving the combination were fatigue, nausea, diarrhea, vomiting, abdominal pain, and arthralgia [56]. Subsequently, in 2020, FDA approved a combination of cetuximab (anti-EGFR monoclonal antibody) and binimetinib for the treatment of patients with adult metastatic colorectal cancer (CRC) harboring a BRAF^V600E^ mutation. This was based on an FDA-approved test, after prior therapy, where the efficacy of treatment was evaluated in a randomized, active-controlled, open-label, multicenter trial (BEACON CRC) on 665 patients with metastatic CRC and BRAF^V600E^ mutation who had shown disease progression after one or two previous treatment regimens [57]. This combination led to a significantly longer OS and a higher ORR response rate than standard therapy. The most common adverse reactions (≥25%) for the combinatorial treatment with encorafenib and cetuximab were fatigue, nausea, diarrhea, acneiform dermatitis, abdominal pain, decreased appetite, arthralgia, and rash [57]. Moreover, unlike the other BRAF inhibitors, this modality could trigger cellular senescence in BRAF^V600E^ melanoma cells [58].

### Mechanisms of anti-BRAF resistance

Potent inhibitors of the BRAF^V600E^ mutant protein, such as dabrafenib, vemurafenib, and trametinib, have produced ORR response rates of 50–60% and shown enhanced PFS and OS rates in patients with BRAF^V600E^ mutations, as compared to dacarbazine [48, 59]. Despite this promising activity, 50% of patients treated with these drugs developed disease progression after several months of treatment. This differential response of patients to these drugs is attributed to BRAF resistance mechanisms. One of the resistance mechanisms identified in the study by Montagut et al. is the expression of CRAF kinases. According to this study, it is possible that increased CRAF protein levels decrease the bioavailability of drugs within mutant cells [60]. These authors also indicated that elevated CRAF protein levels may similarly contribute to primary insensitivity to inhibition in a subset of BRAF-mutant cancer cells [60].

Elevated expression levels of COT represent another resistance mechanism of BRAF. COT is hypothesized to drive resistance to BRAF inhibition predominantly through the reactivation of MAPK signaling [61].

In addition, Shi et al. demonstrated that BRAF^V600E^ amplification results in BRAF^V600E^ overexpression, which is necessary and sufficient for acquired resistance to BRAFi [62].

Yet another mechanism of resistance to BRAFi involves N-RAS upregulation. High levels of activated N-RAS resulting from mutations lead to significant reactivation of the MAPK pathway [63].

Aberrant splicing of BRAF is also known to trigger resistance to BRAFi molecules. A new mechanism of acquired resistance has been identified in patients, where expressed BRAF splice isoforms (such as V600E) dimerize in a RAS-independent manner; the generation of splice variants is likely because of a mutational or epigenetic change affecting BRAF [64].

A notable study conducted by Paraiso et al. addressed the role of PTEN loss in intrinsic resistance to BRAFi. They have shown, for the first time, that loss of PTEN contributes to intrinsic BRAFi resistance via the suppression of BIM-mediated apoptosis [65].

Additional resistance mechanisms include the persistent activation of receptor tyrosine kinases, including platelet-derived growth factor receptor, insulin-like growth factor 1 receptor (IGF-1R), and EGFR. The EGFR/SFK pathway was found to mediate resistance to vemurafenib treatment in BRAF-mutant melanoma, and BRAF and EGFR/SFK inhibition was reported to block the proliferation and invasion of these tumors, providing potentially effective therapeutic options for these patients [66]. As described by Villanueva et al., an increase in IGFR-1R and pAKT levels in a post-relapse human tumor sample is consistent with a role of IGF-1R/PI3K-dependent survival in conferring resistance to BRAFi and could be a plausible explanation of the death of BRAFi-resistant cells upon combined treatment with IGF-1R/PI3K and MEK inhibitors [67].

### Clinical development of anti-BRAF drugs for lung cancer treatment

BRAF is a serine/threonine kinase located inside the cell and is activated by RAS. In turn, BRAF activates downstream kinases, such as MEK and ERK (MAPK). BRAF mutations have been found in half of melanomas, mainly as a V600E mutation [68]. In metastatic non-small cell lung carcinoma (mNSCLC), mutations in BRAF are detected in 2–5% of cases, and the V600E mutant generally occurs in 1–2% of cases. The current data indicate that BRAF mutation in NSCLC is not indicative of any correlation with improved survival or any benefit to chemotherapy in clinical settings, except for the fact that 20–30% of patients with the V600E mutation are non-smokers, whereas the non-V600E subtypes are chain smokers [20, 24, 69–74]. A phase II clinical trial of 36 patients with NSCLC from 19 centers has shown that the presence of BRAF^V600E^ mutation could be associated with increased responsiveness to combination therapy of dabrafenib and trametinib, which act as oral inhibitors of BRAF and MEK, respectively [75].

Increasing our understanding of BRAF biology has enabled the identification of new small molecule inhibitors of the catalytic activity of BRAF.

#### Dabrafenib and trametinib

BRAFi molecules including vemurafenib and dabrafenib as the second line of monotherapy in BRAF-mutant NSCLC showed an ORR of 33–42% and median PFS of 5.5–7.3 months. This combination was further investigated in first (*n* = 36) [75] and second line (*n* = 57) [76] treatments of BRAF^V600E^ positive mNSCLCs. The ORRs were 64% and 63.2%, respectively. The respective median PFS was estimated at 14.6 and 9.7 months. Interim results from the MyPathway study, investigating the efficacy of vemurafenib against BRAF^V600E^ mutation and other BRAF mutations, showed an ORR of 43 (*n* = 14) and 0 (*n* = 7) %, respectively [77].

BRAF and CRAF monomers, heterodimers, and homodimers might improve the efficacy of treatment [78–81]. In fact, BGB-293 is a novel inhibitor of WT BRAF, ARAF, CRAF, EGFR, and BRAF^V600E^. The recommended phase II dose was 40 mg in patients with cancer harboring BRAF or KRAS/NRAS mutations. Of note, there was a partial response in one patient diagnosed with BRAF/MEK inhibitor-näive KRAS-mutated mNSCLC. The major dose-limiting toxicity (DLT) was caused by thrombocytopenia (observed in 13% of patients) [82]. Janku et al. showed that PLX8394 monotherapy or that in combination with a CYP3A4 inhibitor cobicistat increased the efficacy of PLX8394 in refractory solid cancers. The DLT for this combination was observed with respect to elevated levels of aspartate transaminase and alanine transaminase.

### Circulating tumor BRAF as a prognostic marker

The decision to administer targeted therapies in NSCLC is sometimes limited because of unavailable or inadequate biopsies, owing to the difficulty of reaching the tumor region and unknown mutational status in many patients. Liquid biopsy could be a promising way to solve this issue through the detection of mutations in cell-free DNA (cfDNA). BRAF^V600E^ mutations are frequently found in metastatic melanoma. Most of the studies are testing for cfDNA in such clinical settings. Since this mutation is less frequent in lung cancers (1–5%), cfDNA has been studied less frequently in this tumor. However, similarities in responses of EGFR and BRAF in cfDNA toward BRAFi molecules has been observed in two different NSCLC studies [83, 84]. Yang et al. used an innovative competitive allele-specific TaqMan polymerase chain reaction (CastPCR) method to detect driver mutations in cfDNA from 107 lung adenocarcinoma patients. Specificity, sensitivity, concordance values, PPV, and NPV of CastPCR-based detection of EGFR mutations in cfDNA were 94.2% (49/52), 56.4% (31/55), 74.8% (80/107), 91.2% (31/34), and 67.1% (49/73), respectively. Notably, both the specificity value and PPV for p.T790M reached 100% for EGFR. As for BRAF, the CastPCR approach yielded respective values of 28.6% (2/7), 93.0% (93/100), 88.8% (95/107), 22.2% (2/9), and 94.9% (93/98), respectively, which is indicative of good specificity [83]. Similarly, Guilbert et al. observed a good correlation between variations in plasma BRAF mutants in cfDNA and response to BRAF inhibitors in their case study [84]. Ahlborn et al. evaluated the longitudinal tracking of BRAF^V600E^ cfDNA as a marker for responses to BRAFi treatment in non-melanoma tumors. Tumor response was evaluated in half of the patients (8/16), and the median OS and PFS were 15 and 4.8 months, respectively. An increase in longitudinal measurements of BRAF^V600E^ mutant cfDNA indicated disease progression before radiological evaluation, and a reduction of more than 50% of mutations after 4 and 12 weeks of therapy was shown to be significantly associated with prolonged PFS (*p* = 0.003 and *p* = 0.029, respectively) and OS (p = 0.029 and *p* = 0.017, respectively) [85]. Therefore, BRAFi combination therapies showed a ORR response rate of 50% in BRAF^V600E^-mutated non-melanoma tumors.Li et al. demonstrated the reliability of cfDNA against standard immunohistochemistry (IHC) of tissue samples in a study of 190 Chinese patients with lung adenocarcinoma. For the BRAF^V600E^ testing, these authors used the amplification refractory mutation system, based on a fluorescence PCR kit) to analyze the distribution and prognostic role of the mutation. They observed that 5/8 patients with BRAF^V600E^ mutations in IHC matched the plasma DNA samples. The frequency of BRAF^V600E^ mutation in this study of Chinese patients with lung adenocarcinoma was 4.2%, and cfDNA showed good potential for use in BRAF^V600E^ mutational analysis of patients with lung adenocarcinoma [86].

### Ongoing clinical trials

A phase II, open-label clinical study investigating the combined use of debrafenib and trametinib as a second line therapy in 27 patients with BRAF^V600E^-mutated NSCLC is currently ongoing in South Korea (NCT03543306). A further study using this specific combination of drugs has been planned on a larger scale, with 174 participants diagnosed with the same mutated form of cancer (NCT01336634). Investigation of the effect of vemurafenib as a first-line therapeutic for BRAF^V600E^ NSCLC in 60 patients is also imminent (NCT04302025). Use of this drug as both a second- and third-line therapy for the same purpose will also be investigated, using PFS as a primary endpoint in 119 patients. Furthermore, a future phase IV clinical trial is planned to assess the potential adverse effects (AE) of the combination of dabrafenib and trametinib as first-line treatment in 100 patients with BRAF^V600E^-mutated NSCLC (NCT03340506). The full list of ongoing clinical trials investigating BRAFi efficacy in NSCLC treatment is summarized in Table 1.

**Table 1:** Selected ongoing trials with BRAF Inhibitors for NSCLC

Clinical Trial Identifier	Study Design	Intervention/s	Setting	Primary Endpoint	Phase	Status
NCT03645928	75 Participants, Non-Randomized, Parallel Assignment, Open Label	TIL LN-144; TIL LN-144 and if BRAF V600 mutation positive BRAFi or BRAFi with MEKi; TIL LN-144 with pembrolizumab for HNSCC; TIL LN-144 with pembrolizumab for NSCLC; TIL LN-144 single agent for NSCLC	Second or later line	ORR, TEAEs	2	Recruiting
NCT02475213	145 Participants, Non-Randomized, Sequential Assignment, Open Label	Enoblituzumab plus pembrolizumab and BRAFi if V600 mutation positive; enoblituzumab plus MGA012.	First line	AEs	1	Active, not recruiting
NCT03543306	27 Participants, Single group assignment, Non-Randomized, Open label	Dabrafenib plus Trametinib	Second or third line	ORR	2	Recruiting
NCT04302025	60 Participants, Single group assignment, Non-Randomized, Open label	Alectinib; Entrectinib; Vemurafenib; Cobimetinib; Radiotherapy; Chemotherapy	First line	MPR	2	Not yet recruiting
NCT02974725	195 Participants Single group assignment Non Randomized Open Laberl	LXH254x,LTT462, Trametinib,Ribociclib	Second line	AEs	1	Recruiting
NCT01336634	174 Participants, Single group assignment, Non-Radomized, Open label	Dabrafenib plus Trametinib	First line	ORR	2	Active, not recruiting
NCT02314481	119 Participants Single group assignment, Non-randomized Open Label	MPDL3280A, Vemurafenib, Alectinib, Trastuzumab emtansine	Second or third line	PFS	2	Recruiting
NCT03340506	100 Participants, Single group assignment, Interventional Open Label	Dabrafenib plus Trametinib	First line	AEs	4	Recruiting
NCT04190628	27 Participants Sequential Assignment, Non randomized Open Label	ABM-1310	Third and Fourth line therapy	MTD	1	Not yet recruiting
NCT04526782	144 Participants, Interventional, Crossover Assignment Randomized, Open Label	Encorafenib plus Binimetinib	First and Second line therapy	ORR	2	Not yet recruiting
NCT01543698 [1]	179 participants, Single group assignment, Non randomized, Open label	LGX818 in combination with MEK162 in patients with advanced solid tumors	Second or third line	MTD	1b/2	Active, not recruiting

Abbreviations: Adverse Events, AE; Complete Response, CR; Cytokine Release Syndrome, CRS; Dose Limiting Toxicity, DLT; Maximum Tolerated Dose, MTD; Objective Response, OR; Overall Response Rate, ORR; Pharmacodynamics, PD; Partial Response, PR; Progression Free Survival, PFS; Serious Adverse Events, SAEs; Major Pathological Response, MPR; Stable Disease, SD. The information was extracted from www.clinicaltrials.gov.

Previously, a phase II study investigated the use of dabrafenib monotherapy twice daily in combination with trametinib once daily in stage IV NSCLC in patients harboring a V600E mutation (NCT01336634). The main outcomes of this study were the ORR (primary outcome) and OS and PFS (secondary outcomes). Another phase II clinical trial comprising 27 patients with lung cancer has been testing the efficacy of debrafenib combined with trametinib with the same primary and secondary outcomes as defined previously (NCT03543306).

A phase I study was conducted in 145 patients with either melanoma, head and neck cancer, NSCLC, or urothelial carcinoma to identify AEs to the first-line therapy. This was initiated as a two-arm clinical study: one arm was treated with enoblituzumab (anti-B7-H3 monoclonal antibody) and pembrolizumab (anti-PD-1 antibody) along with BRAFi if V600 mutations were detected, whereas the second arm was treated with enoblituzumab and an anti-PD-1 molecule (MGA012) (NCT02475213). A phase II clinical study of 60 patients with NSCLC investigated the use of vemurafenib, alectinib, entrectinib, cobimetinib, radiotherapy, or chemotherapy as first-line therapy. The aim of this study was to identify the major pathological responses of the first-line efficacy of these drugs (NCT04302025).

Another phase II clinical trial that enrolled 119 patients investigated the BRAFi molecules vemurafenib, anti-PD-L1, atezolizumab (MPDL3280A), alectinib, or trastuzumab emtansine (T-DM1). The primary outcome of the study was to observe how PFS differed when patients received each one of the monotherapies (NCT02314481).

A new phase I clinical investigation of 27 participants has been testing the maximum tolerable dose of a small molecule BRAFi (ABM-1310) in advanced solid tumors bearing the BRAF^V600^ mutation (NCT04190628). The novelty of this small molecule is exhibited by its affinity binding to the target receptor in the tumor.

Encorafenib is a BRAFi that targets key enzymes of the MAPK signaling pathway. It has been evaluated in combination with binimetinib (anti-MEK1/2 inhibitors) in a randomized, open-label phase II clinical trial, which enrolled 144 patients with BRAF^V600E^-positive NSCLC, with ORR as the primary outcome (NCT04526782).

Another notable therapy that has been investigated together with BRAFi is the tumor infiltrating lymphocyte (TIL) LN-144. A phase II clinical trial of 75 participants has been evaluating this TIL as a monotherapy or in combination with BRAFi and/or MEKi in patients with the BRAF^V600^ mutation. The study also has additional arms that investigate the efficacy of the LN-144 TIL combined with pembrolizumab or as a monotherapy. The primary outcome of the investigation is in terms of ORR and treatment emergent adverse events, with OS and PFS as secondary outcomes (NCT03645928).

A late phase IV clinical trial is currently investigating the possible AEs of debrafenib in combination with trametinib in 100 patients with NSCLC, melanoma, solid tumors, rare cancers, and high-grade glioma (NCT03340506). It is important to note that targeting BRAF as an anticancer therapy has also been investigated in other solid cancers.

Menzer et al. reported the efficacy of combined BRAFi/MEKi vs. BRAFi monotherapy in a study of 103 patients with metastatic melanoma. Of the 58 patients bearing V600 mutation, the ORR to BRAFi monotherapy and combined BRAFi/MEKi treatment was 27% (6/22) and 56% (20/36), respectively, whereas PFS was 3.7 and 8.0 months, respectively (*p* = 0.002) [87].

The DESCRIBE II trial has evaluated the effectiveness of BRAFi dabrafenib and trametinib in the treatment of BRAF^V600^-mutated metastatic melanoma patients, in a compassionate use setting [88]. This retrospective trial showed substantial clinical activity with dabrafenib plus trametinib in patients with unresectable or metastatic melanoma, similar to what has been assessed in previous prospective controlled trials [89–91]. Furthermore, the analysis of treatment patterns demonstrated the effectiveness of the combinatorial treatment in patients with brain metastases and across lines of therapy with a manageable and well-tolerated safety profile.

## Conclusions

The presence of the BRAF^V600E^ mutation has been found to be associated with increased responsiveness to combined therapy with oral inhibitors of BRAF and MEK. Moreover, the BRAF^V600E^ mutation can occur as a resistance mutation for EGFR-TKI therapy. Currently, the role of BRAF mutations in response to therapy has not yet been understood. The use of liquid biopsy by NGS and targeted real-time PCR could help in rapid identification of BRAF mutational status. The discovery of new technologies that could further improve the sensitivity of detection of mutated BRAF from liquid biopsy would be an exciting frontier in this case, as has been the case for EGFR. FDA approval for anti-BRAF therapy should be further investigated in combination with other drugs that could enhance the immune system, such as checkpoint inhibitors or CAR-T therapies. In fact, these mutations could make the cancers more susceptible to immunotherapies as well as to anti-BRAF therapies. Finally, in addition to the BRAF^V600E^ mutation, novel surrogate biomarkers should be investigated to predict the efficacy of BRAFi for the purpose of stratification of patients in such a way that the best treatment could be given to those who would most likely respond.

## Data Availability

No data were used for this paper.

## References

[1] Association, A. M. (2020) Systemic Therapy for Locally Advanced and Metastatic Non–Small Cell Lung Cancer: A Review 322, 764–774. 10.1001/jama.2019.11058 31454018

[2] Howlader N et al (2019) SEER Cancer statistics review, 1975-2016. National Cancer Institute

[3] Garon EB, Hellmann MD, Rizvi NA, Carcereny E, Leighl NB, Ahn MJ, Eder JP, Balmanoukian AS, Aggarwal C, Horn L, Patnaik A, Gubens M, Ramalingam SS, Felip E, Goldman JW, Scalzo C, Jensen E, Kush DA, Hui R (2019). Five-year overall survival for patients with advanced non-small-cell lung cancer treated with pembrolizumab: results from the phase i KEYNOTE-001 study. J Clin Oncol.

[4] Zhang, J. et al. (2014) Intratumor heterogeneity in localized lung adenocarcinomas delineated by multiregion sequencing. Science (80-. ). doi:10.1126/science.1256930 10.1126/science.1256930 PMC4354858 25301631

[5] Herbst RS, Morgensztern D, Boshoff C (2018). The biology and management of non-small cell lung cancer. Nature.

[6] Gautschi O, Milia J, Cabarrou B, Bluthgen MV, Besse B, Smit EF, Wolf J, Peters S, Früh M, Koeberle D, Oulkhouir Y, Schuler M, Curioni-Fontecedro A, Huret B, Kerjouan M, Michels S, Pall G, Rothschild S, Schmid-Bindert G, Scheffler M, Veillon R, Wannesson L, Diebold J, Zalcman G, Filleron T, Mazières J (2015). Targeted Therapy for Patients with BRAF-Mutant Lung Cancer Results from the European EURAF Cohort. Journal of Thoracic Oncology.

[7] Leicht DT, Balan V, Kaplun A, Singh-Gupta V, Kaplun L, Dobson M, Tzivion G (2007). Raf kinases: function, regulation and role in human cancer. Biochimica et Biophysica Acta - Molecular Cell Research.

[8] Davies H, Bignell GR, Cox C, Stephens P, Edkins S, Clegg S, Teague J, Woffendin H, Garnett MJ, Bottomley W, Davis N, Dicks E, Ewing R, Floyd Y, Gray K, Hall S, Hawes R, Hughes J, Kosmidou V, Menzies A, Mould C, Parker A, Stevens C, Watt S, Hooper S, Wilson R, Jayatilake H, Gusterson BA, Cooper C, Shipley J, Hargrave D, Pritchard-Jones K, Maitland N, Chenevix-Trench G, Riggins GJ, Bigner DD, Palmieri G, Cossu A, Flanagan A, Nicholson A, Ho JWC, Leung SY, Yuen ST, Weber BL, Seigler HF, Darrow TL, Paterson H, Marais R, Marshall CJ, Wooster R, Stratton MR, Futreal PA (2002). Mutations of the BRAF gene in human cancer. Nature.

[9] Wan PTC, Garnett MJ, Roe SM, Lee S, Niculescu-Duvaz D, Good VM, Project CG, Jones CM, Marshall CJ, Springer CJ, Barford D, Marais R (2004). Mechanism of activation of the RAF-ERK signaling pathway by oncogenic mutations of B-RAF. Cell.

[10] Chambard JC, Lefloch R, Pouysségur J, Lenormand P (2007). ERK implication in cell cycle regulation. Biochimica et Biophysica Acta - Molecular Cell Research.

[11] Rustgi AK (2013). BRAF: a driver of the serrated pathway in Colon Cancer. Cancer Cell.

[12] Planchard D, Kim TM, Mazieres J, Quoix E, Riely G, Barlesi F, Souquet PJ, Smit EF, Groen HJM, Kelly RJ, Cho BC, Socinski MA, Pandite L, Nase C, Ma B, D'Amelio A, Mookerjee B, Curtis CM, Johnson BE (2016). Dabrafenib in patients with BRAFV600E-positive advanced non-small-cell lung cancer: a single-arm, multicentre, open-label, phase 2 trial. Lancet Oncol.

[13] Gautschi O, Bluthgen MV, Smit EF, Wolf J, Früh M, Peters S, Schuler M, Zalcman G, Milia J, Mazieres J (2015). Targeted therapy for Braf-mutant lung Cancer: results from the European Euraf cohort study. Ann Oncol.

[14] Hyman DM, Puzanov I, Subbiah V, Faris JE, Chau I, Blay JY, Wolf J, Raje NS, Diamond EL, Hollebecque A, Gervais R*.* (2015) Vemurafenib in multiple nonmelanoma cancers with BRAF V600 mutations. New England J Med 373(8):726–36 10.1056/NEJMoa1502309 PMC4971773 26287849

[15] Odogwu L, Mathieu L, Blumenthal G, Larkins E, Goldberg KB, Griffin N, Bijwaard K, Lee EY, Philip R, Jiang X, Rodriguez L, McKee AE, Keegan P, Pazdur R (2018). FDA approval summary: Dabrafenib and Trametinib for the treatment of metastatic non-small cell lung cancers harboring BRAF V600E mutations. Oncologist.

[16] Shigematsu H, Lin L, Takahashi T, Nomura M, Suzuki M, Wistuba II, Fong KM, Lee H, Toyooka S, Shimizu N, Fujisawa T, Feng Z, Roth JA, Herz J, Minna JD, Gazdar AF (2005). Clinical and biological features associated with epidermal growth factor receptor gene mutations in lung cancers. J Natl Cancer Inst.

[17] Leonetti A, Facchinetti F, Rossi G, Minari R, Conti A, Friboulet L, Tiseo M, Planchard D (2018). BRAF in non-small cell lung cancer (NSCLC): pickaxing another brick in the wall. Cancer Treat Rev.

[18] Pratilas CA, Hanrahan AJ, Halilovic E, Persaud Y, Soh J, Chitale D, Shigematsu H, Yamamoto H, Sawai A, Janakiraman M, Taylor BS, Pao W, Toyooka S, Ladanyi M, Gazdar A, Rosen N, Solit DB (2008). Genetic predictors of MEK dependence in non-small cell lung cancer. Cancer Res.

[19] Kobayashi M et al (2011) Clinical significance of BRAF gene mutations in patients with non-small cell lung cancer. Anticancer Res 22199339

[20] Paik PK, Arcila ME, Fara M, Sima CS, Miller VA, Kris MG, Ladanyi M, Riely GJ (2011). Clinical characteristics of patients with lung adenocarcinomas harboring BRAF mutations. J Clin Oncol.

[21] Samowitz WS, Sweeney C, Herrick J, Albertsen H, Levin TR, Murtaugh MA, Wolff RK, Slattery ML (2005). Poor survival associated with the BRAF V600E mutation in microsatellite-stable colon cancers. Cancer Res.

[22] Viola D, Giannini R, Ugolini C, Biagini A, Romei C, Molinaro E, Agate L, Basolo F, Pinchera A, Elisei R*.* (2011) Low-risk differentiated thyroid cancer with BRAFV600E mutation is more difficult to cure than negative cases: A 5-year follow-up study. J Clin Endocrinol Metab 93(10):3943–9 10.1210/jc.2008-0607 18682506

[23] A., K. et al. (2017) Impact of co-occurring genomic alterations on overall survival of BRAF V600E and non-V600E mutated NSCLC patients: Results of the Network Genomic Medicine. Ann. Oncol. doi:10.1093/annonc/mdx380.003 LK - http://resolver.ebscohost.com/openurl?sid=EMBASE&issn=15698041&id=doi:10.1093%2Fannonc%2Fmdx380.003&atitle=Impact+of+co-occurring+genomic+alterations+on+overall+survival+of+BRAF+V600E+and+non-V600E+mutated+NSCLC+patients%3A+Results+of+the+Network+Genomic+Medicine&stitle=Ann.+Oncol.&title=Annals+of+Oncology&volume=28&issue=&spage=v461&epage=v462&aulast=Kron&aufirst=A.&auinit=A.&aufull=Kron+A.&coden=&isbn=&pages=v461-v462&date=2017&auinit1=A&auinitm=

[24] Marchetti A, Felicioni L, Malatesta S, Grazia Sciarrotta M, Guetti L, Chella A, Viola P, Pullara C, Mucilli F, Buttitta F (2011). Clinical features and outcome of patients with non-small-cell lung cancer harboring BRAF mutations. J Clin Oncol.

[25] De Oliveira Duarte Achcar, R., Nikiforova, M. N. & Yousem, S. A. (2009) Micropapillary Lung adenocarcinoma EGFR, K-ras, and BRAF mutational profile. Am. J. Clin. Pathol. doi:10.1309/AJCPBS85VJEOBPDO 10.1309/AJCPBS85VJEOBPDO 19369630

[26] Yousem SA, Nikiforova M, Nikiforov Y (2008). The histopathology of BRAF-V600E-mutated lung adenocarcinoma. Am J Surg Pathol.

[27] Ikenoue T, Hikiba Y, Kanai F, Aragaki J, Tanaka Y, Imamura J, Imamura T, Ohta M, Ijichi H, Tateishi K, Kawakami T, Matsumura M, Kawabe T, Omata M (2004). Different effects of point mutations within the B-Raf glycine-rich loop in colorectal tumors on mitogen-activated protein/extracellular signal-regulated kinase kinase/extracellular signal-regulated kinase and nuclear factor κB pathway and cellular transfo. Cancer Res.

[28] Loo E, Khalili P, Beuhler K, Siddiqi I, Vasef MA (2018). BRAF V600E mutation across multiple tumor types: correlation between DNA-based sequencing and mutation-specific immunohistochemistry. Appl Immunohistochem Mol Morphol.

[29] Falini B, Martelli MP, Tiacci E (2016). BRAF V600E mutation in hairy cell leukemia: from bench to bedside. Blood.

[30] Cantwell-Dorris ER, O’Leary JJ, Sheils OM (2011). BRAFV600E: implications for carcinogenesis and molecular therapy. Mol Cancer Ther.

[31] Zheng G, Tseng LH, Chen G, Haley L, Illei P, Gocke CD, Eshleman JR, Lin MT (2015). Clinical detection and categorization of uncommon and concomitant mutations involving BRAF. BMC Cancer.

[32] Tian Y, Guo W (2020) A review of the molecular pathways involved in resistance to BRAF inhibitors in patients with advanced-stage melanoma. Med Sci Monit 26:e920957 10.12659/MSM.920957 PMC7169438 32273491

[33] Tang HSC, Chen YC (2015). Insight into molecular dynamics simulation of BRAF(V600E) and potent novel inhibitors for malignant melanoma. Int J Nanomedicine.

[34] Dankner M, Rose AAN, Rajkumar S, Siegel PM, Watson IR (2018). Classifying BRAF alterations in cancer: new rational therapeutic strategies for actionable mutations. Oncogene.

[35] Karoulia Z, Wu Y, Ahmed TA, Xin Q, Bollard J, Krepler C, Wu X, Zhang C, Bollag G, Herlyn M, Fagin JA, Lujambio A, Gavathiotis E, Poulikakos PI (2016). An integrated model of RAF inhibitor action predicts inhibitor activity against oncogenic BRAF signaling. Cancer Cell.

[36] Yao Z, Yaeger R, Rodrik-Outmezguine VS, Tao A, Torres NM, Chang MT, Drosten M, Zhao H, Cecchi F, Hembrough T, Michels J, Baumert H, Miles L, Campbell NM, de Stanchina E, Solit DB, Barbacid M, Taylor BS, Rosen N (2017). Tumours with class 3 BRAF mutants are sensitive to the inhibition of activated RAS. Nature.

[37] Zaman A, Wu W, Bivona TG (2019) Targeting oncogenic braf: past, present, and future. Cancers 11. 10.3390/cancers11081197 10.3390/cancers11081197 PMC6721448 31426419

[38] Jones DTW (2013). Recurrent somatic alterations of FGFR1 and NTRK2 in pilocytic astrocytoma. Nat Genet.

[39] Zehir A, Benayed R, Shah RH, Syed A, Middha S, Kim HR, Srinivasan P, Gao J, Chakravarty D, Devlin SM, Hellmann MD, Barron DA, Schram AM, Hameed M, Dogan S, Ross DS, Hechtman JF, DeLair DF, Yao JJ, Mandelker DL, Cheng DT, Chandramohan R, Mohanty AS, Ptashkin RN, Jayakumaran G, Prasad M, Syed MH, Rema AB, Liu ZY, Nafa K, Borsu L, Sadowska J, Casanova J, Bacares R, Kiecka IJ, Razumova A, Son JB, Stewart L, Baldi T, Mullaney KA, al-Ahmadie H, Vakiani E, Abeshouse AA, Penson AV, Jonsson P, Camacho N, Chang MT, Won HH, Gross BE, Kundra R, Heins ZJ, Chen HW, Phillips S, Zhang H, Wang J, Ochoa A, Wills J, Eubank M, Thomas SB, Gardos SM, Reales DN, Galle J, Durany R, Cambria R, Abida W, Cercek A, Feldman DR, Gounder MM, Hakimi AA, Harding JJ, Iyer G, Janjigian YY, Jordan EJ, Kelly CM, Lowery MA, Morris LGT, Omuro AM, Raj N, Razavi P, Shoushtari AN, Shukla N, Soumerai TE, Varghese AM, Yaeger R, Coleman J, Bochner B, Riely GJ, Saltz LB, Scher HI, Sabbatini PJ, Robson ME, Klimstra DS, Taylor BS, Baselga J, Schultz N, Hyman DM, Arcila ME, Solit DB, Ladanyi M, Berger MF (2017). Mutational landscape of metastatic cancer revealed from prospective clinical sequencing of 10,000 patients. Nat Med.

[40] Bollag G, Hirth P, Tsai J, Zhang J, Ibrahim PN, Cho H, Spevak W, Zhang C, Zhang Y, Habets G, Burton EA, Wong B, Tsang G, West BL, Powell B, Shellooe R, Marimuthu A, Nguyen H, Zhang KYJ, Artis DR, Schlessinger J, Su F, Higgins B, Iyer R, D’Andrea K, Koehler A, Stumm M, Lin PS, Lee RJ, Grippo J, Puzanov I, Kim KB, Ribas A, McArthur GA, Sosman JA, Chapman PB, Flaherty KT, Xu X, Nathanson KL, Nolop K (2010). Clinical efficacy of a RAF inhibitor needs broad target blockade in BRAF-mutant melanoma. Nature.

[41] Chapman PB, Hauschild A, Robert C, Haanen JB, Ascierto P, Larkin J, Dummer R, Garbe C, Testori A, Maio M, Hogg D, Lorigan P, Lebbe C, Jouary T, Schadendorf D, Ribas A, O'Day SJ, Sosman JA, Kirkwood JM, Eggermont AM, Dreno B, Nolop K, Li J, Nelson B, Hou J, Lee RJ, Flaherty KT, McArthur G, BRIM-3 Study Group (2011). Improved survival with Vemurafenib in melanoma with BRAF V600E mutation. N Engl J Med.

[42] Kim G, McKee AE, Ning YM, Hazarika M, Theoret M, Johnson JR, Xu QC, Tang S, Sridhara R, Jiang X, He K, Roscoe D, McGuinn WD, Helms WS, Russell AM, Miksinski SP, Zirkelbach JF, Earp J, Liu Q, Ibrahim A, Justice R, Pazdur R (2014). FDA approval summary: Vemurafenib for treatment of unresectable or metastatic melanoma with the BRAFV600E mutation mutation. Clin Cancer Res.

[43] Oneal PA, Kwitkowski V, Luo L, Shen YL, Subramaniam S, Shord S, Goldberg KB, McKee AE, Kaminskas E, Farrell A, Pazdur R (2018). FDA approval summary: Vemurafenib for the treatment of patients with Erdheim-Chester disease with the BRAF V600 mutation. Oncologist.

[44] Sala E, Mologni L, Truffa S, Gaetano C, Bollag GE, Gambacorti-Passerini C (2008). BRAF silencing by short hairpin RNA or chemical blockade by PLX4032 leads to different responses in melanoma and thyroid carcinoma cells. Mol Cancer Res.

[45] Flaherty K (2008). Cancer Proliferation Gene Discovery Through Functional Genomics. Science (80-. ).

[46] Puszkiel A, Noé G, Bellesoeur A, Kramkimel N, Paludetto MN, Thomas-Schoemann A, Vidal M, Goldwasser F, Chatelut E, Blanchet B (2019). Clinical pharmacokinetics and pharmacodynamics of Dabrafenib. Clin Pharmacokinet.

[47] Kainthla R, Kim KB, Falchook GS (2013). Dabrafenib for treatment of BRAF-mutant melanoma. Pharmgenomics Pers Med.

[48] Hauschild A, Grob JJ, Demidov LV, Jouary T, Gutzmer R, Millward M, Rutkowski P, Blank CU, Miller WH, Kaempgen E, Martín-Algarra S, Karaszewska B, Mauch C, Chiarion-Sileni V, Martin AM, Swann S, Haney P, Mirakhur B, Guckert ME, Goodman V, Chapman PB (2012). Dabrafenib in BRAF-mutated metastatic melanoma: a multicentre, open-label, phase 3 randomised controlled trial. Lancet.

[49] Falchook GS, Long GV, Kurzrock R, Kim KB, Arkenau TH, Brown MP, Hamid O, Infante JR, Millward M, Pavlick AC, O’Day SJ, Blackman SC, Curtis CM, Lebowitz P, Ma B, Ouellet D, Kefford RF (2012). Dabrafenib in patients with melanoma, untreated brain metastases, and other solid tumours: a phase 1 dose-escalation trial. Lancet.

[50] Roskoski R (2018). Targeting oncogenic Raf protein-serine/threonine kinases in human cancers. Pharmacol Res.

[51] Abe H, Kikuchi S, Hayakawa K, Iida T, Nagahashi N, Maeda K, Sakamoto J, Matsumoto N, Miura T, Matsumura K, Seki N, Inaba T, Kawasaki H, Yamaguchi T, Kakefuda R, Nanayama T, Kurachi H, Hori Y, Yoshida T, Kakegawa J, Watanabe Y, Gilmartin AG, Richter MC, Moss KG, Laquerre SG (2011). Discovery of a highly potent and selective MEK inhibitor: GSK1120212 (JTP-74057 DMSO solvate). ACS Med Chem Lett.

[52] Kim KB, Kefford R, Pavlick AC, Infante JR, Ribas A, Sosman JA, Fecher LA, Millward M, McArthur GA, Hwu P, Gonzalez R, Ott PA, Long GV, Gardner OS, Ouellet D, Xu Y, DeMarini DJ, le NT, Patel K, Lewis KD (2013). Phase II study of the MEK1/MEK2 inhibitor trametinib in patients with metastatic BRAF-mutant cutaneous melanoma previously treated with or without a BRAF inhibitor. J Clin Oncol.

[53] Burotto M, Chiou VL, Lee JM, Kohn EC (2014). The MAPK pathway across different malignancies: a new perspective. Cancer.

[54] Savoia P, Fava P, Casoni F, Cremona O (2019) Targeting the ERK signaling pathway in melanoma. Int J Mol Sci 20. 10.3390/ijms20061483 10.3390/ijms20061483 PMC6472057 30934534

[55] Delord JP, Robert C, Nyakas M, McArthur GA, Kudchakar R, Mahipal A, Yamada Y, Sullivan R, Arance A, Kefford RF, Carlino MS, Hidalgo M, Gomez-Roca C, Michel D, Seroutou A, Aslanis V, Caponigro G, Stuart DD, Moutouh-de Parseval L, Demuth T, Dummer R (2017). Phase I dose-escalation and -expansion study of the BRAF inhibitor encorafenib (LGX818) in metastatic BRAF-mutant melanoma. Clin Cancer Res.

[56] Dummer R, Ascierto PA, Gogas HJ, Arance A, Mandala M, Liszkay G, Garbe C, Schadendorf D, Krajsova I, Gutzmer R, Chiarion-Sileni V, Dutriaux C, de Groot JWB, Yamazaki N, Loquai C, Moutouh-de Parseval LA, Pickard MD, Sandor V, Robert C, Flaherty KT (2018). Encorafenib plus binimetinib versus vemurafenib or encorafenib in patients with BRAF-mutant melanoma (COLUMBUS): a multicentre, open-label, randomised phase 3 trial. Lancet Oncol..

[57] Kopetz S, Grothey A, Yaeger R, van Cutsem E, Desai J, Yoshino T, Wasan H, Ciardiello F, Loupakis F, Hong YS, Steeghs N, Guren TK, Arkenau HT, Garcia-Alfonso P, Pfeiffer P, Orlov S, Lonardi S, Elez E, Kim TW, Schellens JHM, Guo C, Krishnan A, Dekervel J, Morris V, Calvo Ferrandiz A, Tarpgaard LS, Braun M, Gollerkeri A, Keir C, Maharry K, Pickard M, Christy-Bittel J, Anderson L, Sandor V, Tabernero J (2019). Encorafenib, Binimetinib, and Cetuximab in BRAF V600E–mutated colorectal Cancer. N Engl J Med.

[58] Li Z, Jiang K, Zhu X, Lin G, Song F, Zhao Y, Piao Y, Liu J, Cheng W, Bi X, Gong P, Song Z, Meng S (2016). Encorafenib (LGX818), a potent BRAF inhibitor, induces senescence accompanied by autophagy in BRAFV600E melanoma cells. Cancer Lett.

[59] Ascierto P (2013). V600E mutation. N Engl J Med.

[60] Montagut C, Sharma SV, Shioda T, McDermott U, Ulman M, Ulkus LE, Dias-Santagata D, Stubbs H, Lee DY, Singh A, Drew L, Haber DA, Settleman J (2008). Elevated CRAF as a potential mechanism of acquired resistance to BRAF inhibition in melanoma. Cancer Res.

[61] Johannessen CM (2011). COT expression predicts resistance to B-RAF inhibition in cancer cell lines.

[62] Shi H (2012). HHS Public Access. 061552.

[63] Hoda Badr, Cindy L. Carmack, Deborah A. Kashy, Massimo Cristofanilli, and T. A R (2011) 基因的改变NIH Public Access. Bone 23, 1–7

[64] Poulikakos PI (2012). HHS Public Access.

[65] Paraiso KHT, Xiang Y, Rebecca VW, Abel EV, Chen YA, Munko AC, Wood E, Fedorenko IV, Sondak VK, Anderson ARA, Ribas A, Palma MD, Nathanson KL, Koomen JM, Messina JL, Smalley KSM (2011). PTEN loss confers BRAF inhibitor resistance to melanoma cells through the suppression of BIM expression. Cancer Res.

[66] Girotti MR, Pedersen M, Sanchez-Laorden B, Viros A, Turajlic S, Niculescu-Duvaz D, Zambon A, Sinclair J, Hayes A, Gore M, Lorigan P, Springer C, Larkin J, Jorgensen C, Marais R (2013). Inhibiting EGF receptor or SRC family kinase signaling overcomes BRAF inhibitor resistance in melanoma. Cancer Discov.

[67] Villanueva J (2011). NIH Public Access.

[68] Ascierto PA (2018). Perspectives in melanoma: Meeting report from the Melanoma Bridge (30 November-2 December, 2017, Naples, Italy). J Transl Med.

[69] Patienten S, Therapie N (2020). PAKT Inhibitor May Promote Better Responses to Abiraterone in mCRPC. Onclive.

[70] Cardarella S, Ogino A, Nishino M, Butaney M, Shen J, Lydon C, Yeap BY, Sholl LM, Johnson BE, Jänne PA (2013). Clinical, pathologic, and biologic features associated with BRAF mutations in non-small cell lung cancer. Clin Cancer Res.

[71] Kris MG, Johnson BE, Berry LD, Kwiatkowski DJ, Iafrate AJ, Wistuba II, Varella-Garcia M, Franklin WA, Aronson SL, Su PF, Shyr Y, Camidge DR, Sequist LV, Glisson BS, Khuri FR, Garon EB, Pao W, Rudin C, Schiller J, Haura EB, Socinski M, Shirai K, Chen H, Giaccone G, Ladanyi M, Kugler K, Minna JD, Bunn PA (2014). Using multiplexed assays of oncogenic drivers in lung cancers to select targeted drugs. JAMA - J Am Med Assoc.

[72] Tissot C, Couraud S, Tanguy R, Bringuier PP, Girard N, Souquet PJ (2016). Clinical characteristics and outcome of patients with lung cancer harboring BRAF mutations. Lung Cancer.

[73] Villaruz LC, Socinski MA, Abberbock S, Berry LD, Johnson BE, Kwiatkowski DJ, Iafrate AJ, Varella-Garcia M, Franklin WA, Camidge DR, Sequist LV, Haura EB, Ladanyi M, Kurland BF, Kugler K, Minna JD, Bunn PA, Kris MG (2015). Clinicopathologic features and outcomes of patients with lung adenocarcinomas harboring BRAF mutations in the lung Cancer mutation consortium. Cancer.

[74] Kinno T, Tsuta K, Shiraishi K, Mizukami T, Suzuki M, Yoshida A, Suzuki K, Asamura H, Furuta K, Kohno T, Kushima R (2004). Clinicopathological features of nonsmall cell lung carcinomas with BRAF mutations - PubMed. Ann Oncol.

[75] Planchard D, Smit EF, Groen HJM, Mazieres J, Besse B, Helland Å, Giannone V, D'Amelio AM, Zhang P, Mookerjee B, Johnson BE (2017). Dabrafenib plus trametinib in patients with previously untreated BRAF V600E -mutant metastatic non-small-cell lung cancer: an open-label, phase 2 trial. Lancet Oncol..

[76] Planchard D, Besse B, Groen HJM, Souquet PJ, Quoix E, Baik CS, Barlesi F, Kim TM, Mazieres J, Novello S, Rigas JR, Upalawanna A, D'Amelio AM, Zhang P, Mookerjee B, Johnson BE (2016). Dabrafenib plus trametinib in patients with previously treated BRAFV600E-mutant metastatic non-small cell lung cancer: an open-label, multicentre phase 2 trial. Lancet Oncol..

[77] Hainsworth JD, Bose R, Sweeney C, Meric-Bernstam F, Hurwitz H, Swanton C, Burris HA, Kurzrock R, Yoo B, Beattie MS, Gupta R, Patel RD, Schulze K, Spigel DR (2017). Targeted therapy for non-small cell lung cancer (NSCLC) with HER2, BRAF, or hedgehog alterations: interim data from MyPathway. J Clin Oncol.

[78] Zhang C, Spevak W, Zhang Y, Burton EA, Ma Y, Habets G, Zhang J, Lin J, Ewing T, Matusow B, Tsang G, Marimuthu A, Cho H, Wu G, Wang W, Fong D, Nguyen H, Shi S, Womack P, Nespi M, Shellooe R, Carias H, Powell B, Light E, Sanftner L, Walters J, Tsai J, West BL, Visor G, Rezaei H, Lin PS, Nolop K, Ibrahim PN, Hirth P, Bollag G (2015). RAF inhibitors that evade paradoxical MAPK pathway activation. Nature.

[79] Yao Z, Torres NM, Tao A, Gao Y, Luo L, Li Q, de Stanchina E, Abdel-Wahab O, Solit DB, Poulikakos PI, Rosen N (2015). BRAF mutants evade ERK-dependent feedback by different mechanisms that determine their sensitivity to pharmacologic inhibition. Cancer Cell.

[80] Peng S (2015). Bin *et al.* inhibition of RAF isoforms and active dimers by LY3009120 leads to anti-tumor activities in RAS or BRAF mutant cancers. Cancer Cell.

[81] Girotti MR, Lopes F, Preece N, Niculescu-Duvaz D, Zambon A, Davies L, Whittaker S, Saturno G, Viros A, Pedersen M, Suijkerbuijk BMJM, Menard D, McLeary R, Johnson L, Fish L, Ejiama S, Sanchez-Laorden B, Hohloch J, Carragher N, Macleod K, Ashton G, Marusiak AA, Fusi A, Brognard J, Frame M, Lorigan P, Marais R, Springer C (2015). Paradox-breaking RAF inhibitors that also target SRC are effective in drug-resistant BRAF mutant melanoma. Cancer Cell.

[82] Wu JY, Yu CJ, Chang YC, Yang CH, Shih JY, Yang PC (2011). Effectiveness of tyrosine kinase inhibitors on ‘uncommon’ epidermal growth factor receptor mutations of unknown clinical significance in non-small cell lung cancer. Clin Cancer Res.

[83] Yang Y, Shen X, Li R, Shen J, Zhang H, Yu L, Liu B, Wang L (2017). The detection and significance of EGFR and BRAF in cell-free DNA of peripheral blood in NSCLC. Oncotarget.

[84] Guibert N, Pradines A, Casanova A, Farella M, Keller L, Soria JC, Favre G, Mazières J (2016). Detection and monitoring of the BRAF mutation in circulating tumor cells and circulating tumor DNA in BRAF-mutated lung adenocarcinoma. J Thorac Oncol.

[85] Ahlborn LB, Tuxen IV, Mouliere F, Kinalis S, Schmidt AY, Rohrberg KS, Santoni-Rugiu E, Nielsen FC, Lassen U, Yde CW, Oestrup O, Mau-Sorensen M (2018). Circulating tumor DNA as a marker of treatment response in BRAF V600E mutated non-melanoma solid tumors. Oncotarget.

[86] Li Z, Jiang L, Bai H, Wang Z, Zhao J, Duan J, Yang X, An T, Wang J (2015). Prevalence and clinical significance of BRAF V600E in Chinese patients with lung adenocarcinoma. Thorac Cancer.

[87] Menzer C, Menzies AM, Carlino MS, Reijers I, Groen EJ, Eigentler T, de Groot JWB, van der Veldt AAM, Johnson DB, Meiss F, Schlaak M, Schilling B, Westgeest HM, Gutzmer R, Pföhler C, Meier F, Zimmer L, Suijkerbuijk KPM, Haalck T, Thoms KM, Herbschleb K, Leichsenring J, Menzer A, Kopp-Schneider A, Long GV, Kefford R, Enk A, Blank CU, Hassel JC (2019). Targeted therapy in advanced melanoma with rare BRAF mutations. J Clin Oncol.

[88] Chalmers A, Cannon L, Akerley W (2019). Adverse event Management in Patients with BRAF V600E-mutant non-small cell lung Cancer treated with Dabrafenib plus Trametinib. Oncologist.

[89] Long GV, Stroyakovskiy D, Gogas H, Levchenko E, de Braud F, Larkin J, Garbe C, Jouary T, Hauschild A, Grob JJ, Chiarion Sileni V, Lebbe C, Mandalà M, Millward M, Arance A, Bondarenko I, Haanen JB, Hansson J, Utikal J, Ferraresi V, Kovalenko N, Mohr P, Probachai V, Schadendorf D, Nathan P, Robert C, Ribas A, DeMarini DJ, Irani JG, Casey M, Ouellet D, Martin AM, Le N, Patel K, Flaherty K (2014) Combined BRAF and MEK inhibition versus BRAF inhibition alone inmelanoma. N Engl J Med 371(20):1877–88 10.1056/NEJMoa1406037 25265492

[90] Schadendorf D, Amonkar MM, Stroyakovskiy D, Levchenko E, Gogas H, de Braud F, Grob JJ, Bondarenko I, Garbe C, Lebbe C, Larkin J, Chiarion- Sileni V, Millward M, Arance A, Mandalà M, Flaherty KT, Nathan P, Ribas A, Robert C, Casey M, DeMarini DJ, Irani JG, Aktan G, Long GV (2015) Healthrelated quality of life impact in a randomised phase III study of the combination of dabrafenib and trametinib versus dabrafenib monotherapy in patients with BRAF V600 metastatic melanoma. Eur J Cancer 51(7):833–40 10.1016/j.ejca.2015.03.004 25794603

[91] Flaherty KT, Infante JR, Daud A, Gonzalez R, Kefford RF, Sosman J, Hamid O, Schuchter L, Cebon J, Ibrahim N, Kudchadkar R, Burris HA, Falchook G, Algazi A, Lewis K, Long GV, Puzanov I, Lebowitz P, Singh A, Little S, Sun P, Allred A, Ouellet D, Kim KB, Patel K, Weber J (2012). Combined BRAF and MEK inhibition in melanoma with BRAF V600 mutations. N Engl J Med.

